# Clinicopathological significance of loss of Y chromosome in male meningiomas

**DOI:** 10.1002/path.70040

**Published:** 2026-02-19

**Authors:** Maki Sakaguchi, Masafumi Horie, Yukinobu Ito, Shingo Tanaka, Hiroko Ikeda, Mitsutoshi Nakada, Akihiko Yoshizawa, Daichi Maeda

**Affiliations:** ^1^ Department of Molecular and Cellular Pathology, Graduate School of Medical Sciences Kanazawa University Kanazawa Japan; ^2^ Department of Diagnostic Pathology Nara Medical University Kashihara Japan; ^3^ Division of Molecular and Genomic Pathology, Department of Pathology Kobe University Graduate School of Medicine Kobe Japan; ^4^ Department of Neurosurgery, Graduate School of Medical Sciences Kanazawa University Kanazawa Japan; ^5^ Department of Diagnostic Pathology Kanazawa University Hospital Kanazawa Japan; ^6^ The Garvan Institute of Medical Research Darlinghurst New South Wales Australia

**Keywords:** male meningioma, loss of Y chromosome (LOY), merlin, droplet digital polymerase chain reaction, multiplex ligation‐dependent probe amplification, RNA *in situ* hybridization, Xenium

## Abstract

Male meningiomas, comprising approximately 30% of all meningiomas, are more frequently high‐grade and associated with poorer clinical outcomes compared to their female counterparts. Although Y chromosome alterations have been studied in various male‐predominant tumors, a limited number of studies have evaluated their role in meningiomas. To evaluate the clinicopathological significance of Y chromosome loss in male meningiomas, we assessed the frequency of loss of the Y chromosome (LOY) using droplet digital polymerase chain reaction in combination with multiplex ligation‐dependent probe amplification on tumor DNA from 93 male meningioma samples. LOY, detected in nine cases (9.7%), was significantly associated with a higher World Health Organization tumor grade (grade 2: 55.6% versus 14.3%; grade 1: 44.4% versus 85.7%; *p* = 0.009) and loss of the NF2 gene‐encoded protein, moesin‐ezrin‐radixin‐like protein (merlin) (loss: 88.9% versus 50.0%; retained: 11.1% versus 50.0%; *p* = 0.035). RNA *in situ* hybridization targeting *KDM5D* on formalin‐fixed paraffin‐embedded tissue sections demonstrated a sensitivity of 100% (9/9) and a specificity of 76.2% (64/84) for LOY detection, supporting its utility as a screening modality. Moreover, spatial transcriptomic analysis revealed significant differences in the expression of genes associated with epithelial–mesenchymal transition and extracellular matrix organization between LOY and non‐LOY meningioma tumor cells. Our findings emphasize the presence of atypical pathological features and distinct transcriptional profiles in LOY‐associated meningiomas. © 2026 The Author(s). *The Journal of Pathology* published by John Wiley & Sons Ltd on behalf of The Pathological Society of Great Britain and Ireland.

## Introduction

Meningioma is the most common primary intracranial tumor, accounting for approximately 40% of all primary brain tumors and 56% of nonmalignant primary brain tumors [1]. Although most meningiomas are classified as grade 1 (benign), up to 20% are designated as atypical (grade 2) or anaplastic (grade 3) according to the 2021 World Health Organization (WHO) classification [1, 2]. The incidence of meningioma varies with sex, age, ethnicity, and race, with a higher prevalence observed among females, older individuals, and non‐Hispanic Black populations [3]. Although male meningiomas, accounting for approximately 30% of all meningiomas, are more frequently high‐grade and associated with poorer prognoses, compared to their female counterparts, clinicopathological studies focusing specifically on male meningiomas remain limited [3, 4]. Furthermore, comprehensive genomic analyses investigating sex‐specific differences in meningioma biology are currently lacking.

In recent years, loss of the Y chromosome (LOY) in peripheral blood cells (PBCs) has garnered considerable attention due to its association with increased mortality and elevated risk for a range of age‐related diseases, including hematologic and solid malignancies, diabetes mellitus, cardiovascular disorders, and Alzheimer's disease. [5, 6, 7]. Investigations of LOY in solid tumors have been limited, with most using fluorescence *in situ* hybridization (FISH) to assess renal tumors, such as papillary renal cell carcinoma and chromophobe renal cell carcinoma, and esophageal squamous cell carcinoma, adenocarcinoma, and urothelial carcinoma of the urinary bladder [8, 9, 10, 11]. Qi *et al* reported that, in tumors, complete LOY is more prevalent than partial LOY, the latter defined as the loss of at least one segment of the Y chromosome with retaining wild type (WT) copy number in the remaining regions [12]. We recently performed whole‐exome sequencing in a case of sporadic multiple meningiomas and found that copy‐number loss on chromosomes 22q and complete LOY were recurrent abnormalities in the two largest tumors [13]. Despite these observations, the prevalence and clinicopathological relevance of complete LOY in male meningiomas remain uncertain.

In meningiomas, previous studies identified deletions of chromosome 22 or mutations in the tumor suppressor *NF2* gene, located on chromosome 22q12.2, in up to 60% of sporadic cases [14]. Meningiomas harboring *NF2* alterations are predisposed to higher histological grades and exhibit increased biological aggressiveness, largely attributed to genomic instability [15, 16]. In contrast, NF2‐WT meningiomas frequently possess distinct driver mutations in genes, such as *TRAF7*, *KLF4*, *AKT1*, *SMO*, *SUFU*, *PRKAR1A*, *PIK3CA*, and *POLR2A* [17, 18, 19]. Moesin‐ezrin‐radixin‐like protein (merlin), the protein product of the *NF2* gene, functions as a tumor suppressor and plays a critical role in meningioma tumorigenesis [20].

We evaluated the biological significance of LOY in male meningiomas. In a cohort of 93 cases, we assessed the frequency of LOY, its clinicopathological relevance, and its association with underlying genetic alterations, including loss of merlin expression. To overcome the limitations of conventional FISH, we used droplet digital polymerase chain reaction (ddPCR), a highly sensitive technique, for initial screening and validated the findings using multiplex ligation‐dependent probe amplification (MLPA) to enhance analytical accuracy and to evaluate both the short and long arms of the Y chromosome. Additionally, we assessed the detectability of LOY using RNA *in situ* hybridization. Spatial transcriptome analyses were also performed to characterize the molecular and spatial features associated with LOY.

## Materials and methods

### Ethics approval and consent to participate

The use of human subject material was performed in accordance with the World Medical Association Declaration of Helsinki [21], and the study protocol was approved by the Kanazawa University Medical Ethics Committee (No. 72682‐1). Written informed consent was obtained from all participants.

### Patient cohort and sample acquisition

In total, 93 newly diagnosed meningioma samples were collected from 85 male patients who underwent surgical resection at Kanazawa University Hospital between 2015 and 2023. This cohort included 12 samples derived from synchronous multiple lesions in four patients. All surgical specimens were fixed in 4% paraformaldehyde and processed into formalin‐fixed paraffin‐embedded (FFPE) tissue blocks. Full tissue sections, 4 μm in thickness, were prepared for hematoxylin and eosin (H&E) staining. Tissue microarrays (TMAs) were constructed by extracting a 2‐mm‐diameter core from a representative region of each FFPE block. TMA‐based analysis was conducted for 92 cases; one case, which lacked sufficient tissue for TMA construction, was analyzed using whole‐slide sections for immunohistochemistry and *in situ* hybridization. For spatial transcriptomic analysis using the Xenium platform, nine representative cases were selected: three cases of LOY (Nos. 1–3) and six cases of non‐LOY (Nos. 4–9) meningiomas. These samples were included in a separate TMA for Xenium analysis. The histological subtypes of the selected cases were as follows (based on quality‐controlled samples): No. 1 – atypical; Nos. 3 and 9 – transitional; Nos. 4, 5, 6, and 8 – meningothelial; No. 7 – angiomatous. Clinical data were retrieved from electronic medical records. Magnetic resonance imaging (MRI) was evaluated by a neurosurgeon (TS).

### Histopathological and immunohistochemical analysis

Histopathological analysis was performed on all specimens by experienced pathologists (MS and HI). Based on the 2021 WHO diagnostic criteria for CNS tumors, 76 samples (81.7%) were classified as WHO grade 1 and 17 samples (18.3%) as WHO grade 2 [2]. For all cases, the diagnostic features of atypical meningioma were assessed individually, including mitotic activity, brain invasion, high cellularity, small cells with a high nuclear–cytoplasmic ratio, prominent nucleoli, sheeting architecture, and spontaneous necrosis.

Immunohistochemical staining was performed on all 93 samples using standard protocols on the Ventana Benchmark Autostainer (F. Hoffmann‐La Roche Ltd., Basel, Switzerland). The following primary antibodies were used: anti‐Merlin (clone D3S3W, Catalog No.: 12888, diluted 1:50; Cell Signaling Technologies Inc., Danvers, MA, USA), anti‐MTAP (clone H0004507‐M01, diluted 1:100; Abnova GmbH, Heidelberg, Germany), and anti‐Ki‐67 (clone IR62661‐2, ready‐to‐use formulation; DAKO, Glostrup, Denmark). Appropriate positive and negative controls were included in each run to validate staining specificity and quality.

Merlin expression was considered retained when tumor cells exhibited positive staining, regardless of intensity, and classified as lost when tumor cells were negative in the presence of a positive internal control. Methylthioadenosine phosphorylase (MTAP) expression was deemed retained if strong nuclear and cytoplasmic staining was observed and classified as lost when cytoplasmic staining was completely absent or weaker than that of the internal control [22, 23].

### DNA extraction

Genomic DNA was extracted from paraffin‐embedded sections of 93 male meningioma samples using the DNeasy Blood & Tissue Kit (Qiagen, Hilden, Germany) and quantified using a NanoDrop 2000 spectrophotometer (Thermo Fisher Scientific, Waltham, MA, USA). Samples with low tumor cell content were manually macrodissected to enrich for tumor tissue.

### Sanger sequencing

Sanger sequencing was used to evaluate *TERT* promoter mutations. Genomic DNA was amplified using AmpliTaq Gold 360 DNA Polymerase (Thermo Fisher Scientific), and PCR products were purified with the QIAEX II Gel Extraction Kit (Qiagen). PCR was performed for 45 cycles, each consisting of denaturation at 94 °C for 60 s, annealing at 60 °C for 30 s, and extension at 72°C for 30 s. Sequencing reactions were carried out using the BigDye Terminator version 3.1 Cycle Sequencing Kit (Thermo Fisher Scientific) and analyzed on the Applied Biosystems 3500xl DNA Sequencer (Thermo Fisher Scientific). The primers used for amplification were *TERT* forward (5’‐CACCCGTCCTGCCCCTTCACCT‐3’) and *TERT* reverse (5’‐GGCTTCCCACGTGCGCAGCAGGA‐3’).

### Droplet digital PCR (ddPCR)

Genomic DNA extracted from meningioma samples was diluted to a concentration of 30 ng/μl. ddPCR was performed using the Absolute Q ddPCR System (Thermo Fisher Scientific). A TaqMan gene copy‐number assay targeting the *SRY* gene (FAM‐labeled, dHsaCP2500472, Bio‐Rad Laboratories, Hercules, CA, USA), located on the short arm of the Y chromosome, was used. Primers and probe for the reference gene *AP3B1* were designed using Primer Express Software (Thermo Fisher Scientific) with the following sequences: forward primer, 5’‐GGCAACAGGGACACACTTGA‐3’; reverse primer, 5’‐TGGTGCTATGTCCACAAAGAGAA‐3’; and VIC‐labeled probe, 5’‐AGCCAGCAAAATCTCAAAAGAAGCCACA‐3’. Each PCR reaction mixture contained 60 ng genomic DNA, 5× Master Mix, 900 nmol/l of SRY primers, 900 nmol/l of AP3B1 primers, and 200 nmol/l of each probe. A total of 9 μl of the PCR mix was dispensed into the wells of the Microfluidic Array Partitioning plate, followed by the addition of 15 μl of isolation buffer. The plates were sealed with gasket caps and loaded into the Absolute Q system. PCR was carried out under the following thermal cycling conditions: initial activation at 95 °C for 10 min, followed by 40 cycles of 96 °C for 5 s and 60 °C for 30 s. Data acquisition and analysis were performed using QuantStudio 6 software (Thermo Fisher Scientific). The copy number of the *SRY* gene was calculated using *AP3B1* as the reference gene, with its copy number set at 2. Considering that the samples contained a significant number of nonneoplastic cells, we empirically set a cutoff threshold of 0.5 for LOY screening.

### Multiplex ligation‐dependent probe amplification

All candidate cases exhibiting a ddPCR‐derived copy number below 0.5, together with eight cases exceeding 0.5, were analyzed using MLPA with the SALSA MLPA Probemix P360‐B2 (Y‐Chromosome) (MRC‐Holland). The P360 probemix comprises 43 probes targeting the male‐specific region of the Y chromosome and 12 autosomal reference probes for normalization, as specified by the manufacturer (supplementary material, Table S1). For each MLPA run, in addition to the tumor samples under investigation, at least three normal control DNA samples and WT DNA were included to ensure data accuracy. Reference DNAs were extracted from normal (nontumorous) FFPE brain tissue resected for cerebral hemorrhage, arteriovenous malformation, or metastatic carcinoma. MLPA reactions were performed following the manufacturer's standard protocol with minor laboratory optimizations. In brief, 100 ng genomic DNA per reaction was denatured at 98 °C for 5 min and immediately hybridized with 1.5 μl of the P360 probemix in a total hybridization volume of 3.0 μl buffer at 60 °C for 16–20 h. Ligation was subsequently carried out using Ligase‐65 (MRC‐Holland) for 15 min at 54 °C, followed by enzyme inactivation at 98 °C for 5 min. PCR amplification was conducted using SALSA polymerase and MLPA‐specific primers under the following cycling conditions: 35 cycles of 95 °C for 30 s, 60 °C for 30 s, and 72 °C for 60 s. PCR products were separated by capillary electrophoresis on a DS3000 Compact CE Sequencer (HITACHI) using the GeneScan 500LIZ dye Size Standard (Applied Biosystems, Catalog No.: 4322682).

Electropherograms were analyzed using Coffalyser.Net software (MRC‐Holland). Peak heights were exported, and probe signals were normalized following the manufacturer's recommended two‐step procedure: within‐sample normalization to the median of the autosomal reference probes, followed by between‐sample normalization to a set of male reference DNAs processed on the same plate. The probe ratio (test/reference) was calculated for each probe, and the final ratio for each gene was determined as the average value across multiple probes, normalized such that the ratio of WT DNA was set to 1.

In accordance with a previous report, a final ratio of ≤ 0.8 was interpreted as indicative of copy‐number loss, and a ratio of ≥ 1.2 as indicative of copy‐number gain [24]. Complete LOY was defined as the presence of copy‐number loss across all 20 Y‐chromosome genes analyzed.

### RNA *in situ* hybridization

Among the limited number of Y chromosome‐encoded genes expressed in adult tissues – such as *RPS4Y1*, *DDX3Y*, *TXLNGY*, *KDM5D*, and *EIF1AY* – we selected *KDM5D* for RNA *in situ* hybridization analysis due to the commercial availability of a validated probe. *In situ* hybridization of *KDM5D* transcripts was performed on paraffin‐embedded TMA sections using the RNAscope assay with Probe‐Hs‐KDM5D (Catalog No.: 558161, Advanced Cell Diagnostics, Newark, CA, USA) and the RNAscope 2.5 HD Reagent Kit (Catalog No.: 322350, Advanced Cell Diagnostics). TMA sections (5 μm thick) were deparaffinized, subjected to heat‐induced epitope retrieval in a pretreatment reagent for 15 min, and subsequently digested with protease at 40 °C for 30 min. Hybridization with Probe‐Hs‐KDM5D was carried out for 2 h at 40 °C. Signal detection was performed using 3,3’‐diaminobenzidine, and nuclei were counterstained with hematoxylin. Probe‐Hs‐*PPIB* (Catalog No.: 313901, Advanced Cell Diagnostics) was used as a positive control, and a female meningioma sample served as a negative control. RNAscope staining was interpreted as positive when one or more dots per tumor cell were observed, with or without dot clusters. Staining was considered negative when no signal or fewer than one dot per 10 tumor cells was detected.

### *In situ* gene expression profiling

Xenium *in situ* expression analysis was performed using Xenium slides and sample preparation reagents (PN‐1000742, 10x Genomics, Pleasanton, CA, USA). FFPE tissues were sectioned at 5‐μm thickness and placed on Xenium slides. Deparaffinization and decrosslinking were carried out following the manufacturer's protocol (CG000580, 10x Genomics). Slide preparation followed the user guide for Xenium Prime *In Situ* Gene Expression with Optical Cell Segmentation (CG000760, 10x Genomics). The Xenium Human 5K Pan‐Tissue & Pathways Panel was used for hybridization. Imaging and decoding were conducted using the Xenium Analyzer (on‐board analysis: version xenium‐3.1.0.4; software: version 3.1.0.0, 10x Genomics). Image data and expression profiles were further evaluated using Xenium Explorer (version 3.2.0, 10x Genomics). Following the Xenium run, H&E staining were performed on the same slide.

### Analysis of spatial transcriptome data

Spatial transcriptomic data generated by the Xenium Analyzer were further processed and analyzed using the Seurat package (version 4.4.0; https://satijalab.org/seurat/articles/install_v5.html). Data normalization was performed using the *SCTransform* function. Clustering analysis was carried out based on the first 30 principal components (PCs) using the *FindNeighbors* and *FindClusters* functions, with the resolution parameter set to 0.3. Furthermore, Uniform Manifold Approximation and Projection (UMAP) was performed using the first 30 PCs. Differentially expressed genes for each cluster were identified using the *FindAllMarkers* function. Deconvolution analysis was performed using the RCTD method from the spacexr package (version 2.2.1) (https://github.com/dmcable/spacexr) [25], with reference single‐cell RNA‐seq data obtained from dataset GSE183656 (https://www.ncbi.nlm.nih.gov/geo/query/acc.cgi?acc=GSE183656). Pathway enrichment analysis was conducted using Enrichr [26, 27, 28, 29].

### Statistical analyses

The Mann–Whitney *U* test and Fisher's exact test were used to assess associations between clinical variables and the presence of LOY. Five‐year progression‐free survival (PFS) was estimated using the Kaplan–Meier method, and survival curves were compared using a nonstratified log‐rank test. A *p* value < 0.05 was considered statistically significant. Statistical analyses were performed using EZR (Saitama Medical Center, Jichi Medical University, Saitama, Japan) [30].

## Results

### LOY in male meningioma

In total, 93 meningioma samples were obtained from 85 male patients, including 12 samples from four patients with synchronous multiple lesions. All patients were aged ≥31 years, with a median age of 67 years (range: 31–88 years) (Table 1). Tumor localization was classified as anterior skull base (*n* = 24), middle skull base (*n* = 17), posterior skull base (*n* = 12), and non–skull base regions (*n* = 40). In total, 13 patients experienced tumor recurrence; however, no meningioma‐related deaths were recorded during the study period. The median postoperative follow‐up duration was 43.5 months (range: 4.3–118.2 months).

**Table 1:** Comparison of clinicopathological characteristics between LOY and non‐LOY meningiomas.

		LOY+	LOY−	Total	*p* value
*n*		9 (9.7)	84 (90.3)	93	
Age		67 [53, 86]	67 [31, 88]	67 [31, 88]	0.373
Location	Anterior skull base	0 (0)	24 (28.6)	24 (25.8)	0.204
	Middle skull base	2 (22.2)	15 (17.9)	17 (18.3)	
	Posterior skull base	1 (11.1)	11 (13.1)	12 (12.9)	
	Non skull base	6 (66.7)	34 (40.5)	40 (43.0)	
Tumor size (cm)		4.0 [2.7, 6.1]	3.5 [1.1, 6.8]		0.151
Extent of resection	Total resection	5 (55.6)	45 (53.6)	50 (53.8)	1
	Partial resection	4 (44.4)	39 (46.4)	43 (46.2)	
Adjuvant radiotherapy	−	6 (66.7)	73 (86.9)	79 (84.9)	0.132
	+	3 (33.3)	11 (13.1)	14 (15.1)	
Recurrent	−	7 (77.8)	72 (86.7)	79 (85.9)	0.610
	+	2 (22.2)	11 (13.3)	13 (14.1)	
Subtype (grade 1)	Meningothelial	2 (33.3)	33 (38.5)	35 (37.6)	0.947
	Transitional	2 (20.0)	26 (32.1)	28 (30.1)	
	Fibrous	0 (6.7)	7 (7.7)	7 (7.5)	
	Angiomatous	0 (0.0)	5 (6.4)	5 (5.4)	
	Microcystic	0 (0.0)	1 (1.3)	1 (1.1)	
WHO grade	1	4 (44.4)	72 (85.7)	76 (81.7)	0.009*
	2	5 (55.6)	12 (14.3)	17 (18.3)	
MTAP IHC	Retained	15 (100)	78 (100)	93 (100)	NA
	Lost	0	0	0 (0)	
Merlin IHC	Retained	1 (11.1)	42 (50.0)	43 (46.2)	0.035*
	Lost	8 (88.9)	42 (50.0)	50 (53.3)	
Ki‐67 labeling index (%)		2.2 [0.6, 6.3]	1.4 [0.0, 14.2]	1.3 [0.0, 14.2]	0.308

Values are shown as *n* (%) or median [range].

LOY, loss of Y chromosome; WHO, World Health Organization; MTAP, methylthioadenosine phosphorylase; IHC, immunohistochemistry; NA, not available.

* *p* < 0.05.

**Figure 1:**
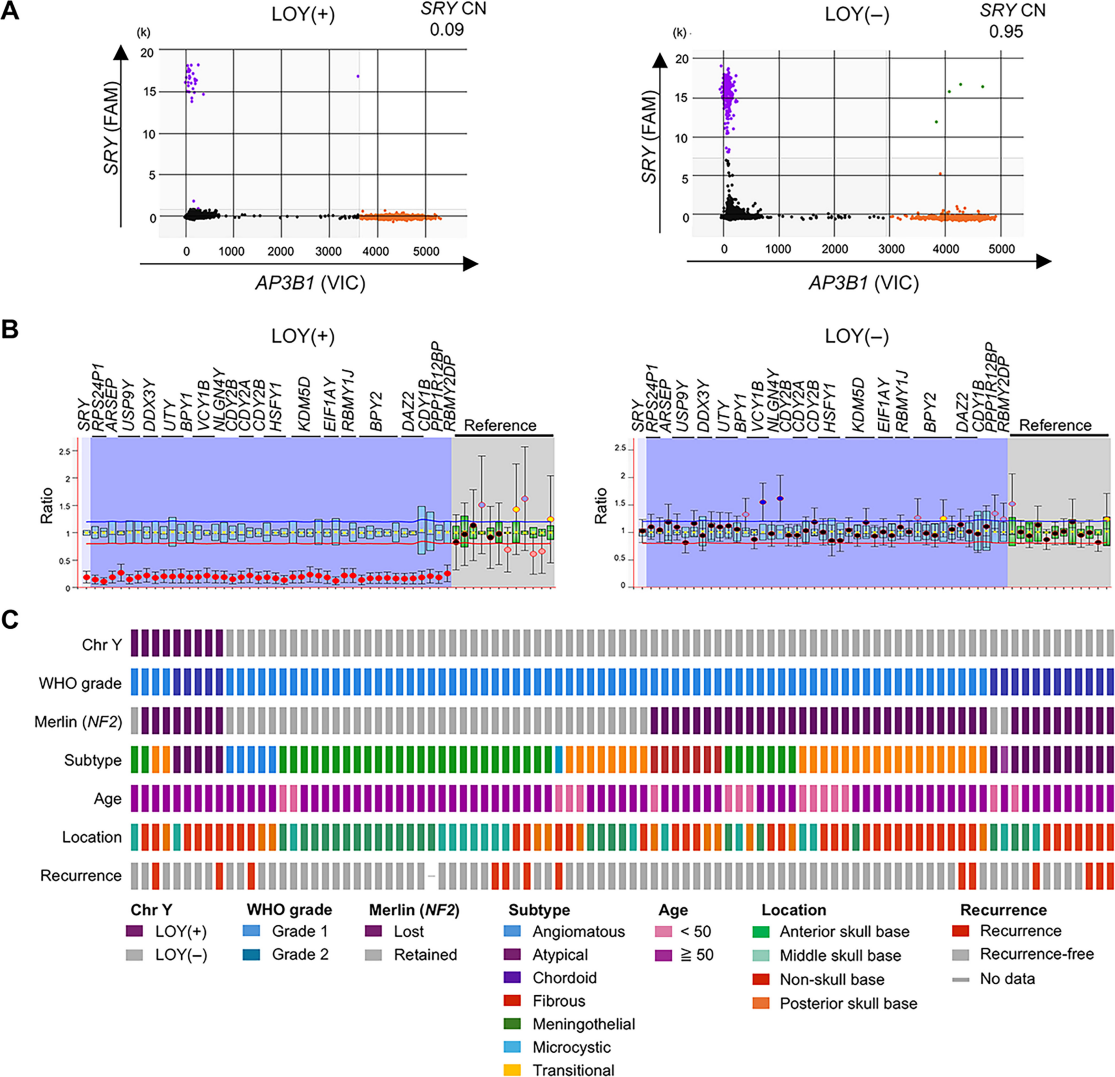
Detection of Y chromosome loss and clinicopathological overview. (A) Two‐dimensional cluster plot of ddPCR targeting the *AP3B1* reference gene and the *SRY* gene on the Y chromosome. Representative plots for cases with loss of the Y chromosome (LOY+) and without LOY (LOY−) are shown. Each dot represents a droplet categorized by fluorescence amplitude: *SRY* (purple), *AP3B1* (orange), both targets (green), or neither (black). The *x*‐axis indicates fluorescence amplitude for the VIC fluorophore (*AP3B1*), and the *y*‐axis indicates fluorescence amplitude for the FAM fluorophore (*SRY*). (B) MLPA using the P360 probe mix includes 43 probes targeting the male‐specific region of the Y chromosome and 12 autosomal reference probes for normalization. Representative cases of LOY+ and LOY−. A probe ratio between 0.8 and 1.2 is interpreted as a normal copy number, whereas a ratio of 0.8 or less is considered indicative of copy‐number loss. (C) Summary of Y chromosome status and clinicopathological characteristics in 93 male meningioma cases. LOY was identified in 9.7% of cases.

**Figure 2:**
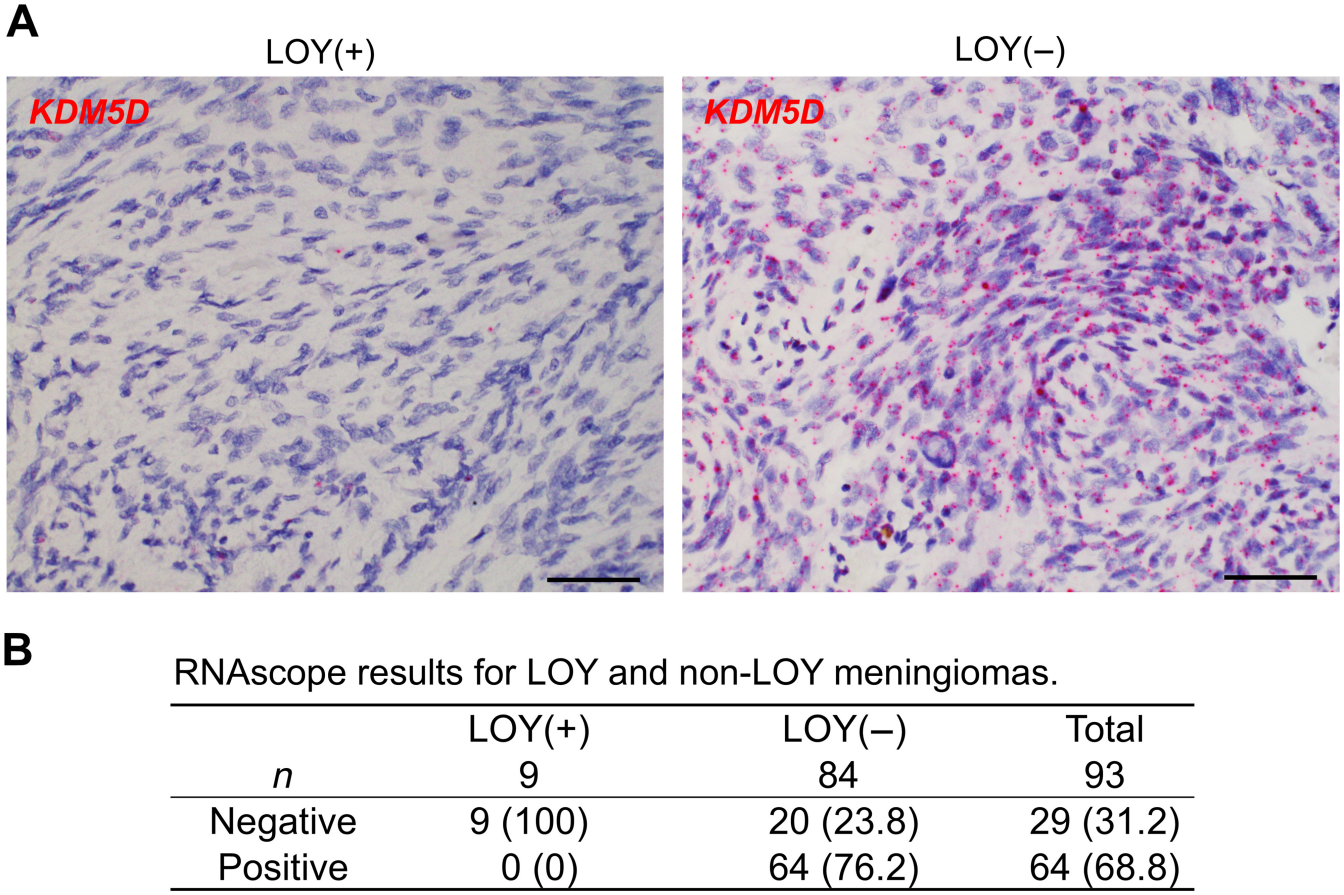
RNAscope‐based detection of LOY. (A) RNAscope analysis targeting *KDM5D*, a Y chromosome–linked gene, in LOY‐positive (left) and LOY‐negative (right) meningioma samples. Scale bar, 50 μm. (B) Corresponding RNAscope results for LOY and non‐LOY meningiomas, as determined by the combined application of ddPCR and MLPA.

In LOY screening, ddPCR targeting the *SRY* gene on the Y chromosome identified 15 of 93 cases (16.1%) in which tumor tissue DNA samples exhibited copy‐number values below the cutoff threshold of 0.5 (Figure 1A). Subsequent MLPA analysis was performed on these 15 candidate LOY cases, of which nine were identified as complete LOY (Figure 1B,C; supplementary material, Figure S1). The remaining six cases exhibited partial or focal deletions ranging from 10 to 60% of Y‐chromosomal loci. Localized or segmental copy‐number losses outside the AZFc region – known to be deleted in approximately 30% of Japanese men [31] – were detected in five cases, two of which showed loss of *SRY*. Complete LOY was not demonstrated by MLPA in any of the eight cases with ddPCR‐derived copy numbers exceeding 0.5. In parallel, RNAscope analysis of *KDM5D*, a gene product encoded on the Y chromosome, provided supportive evidence for detecting LOY in FFPE tissue sections (Figure 2A). Among the cohort, 29 cases (31.2%) exhibited negative *KDM5D* expression, characterized by the absence or near absence of signal, whereas 64 cases (68.8%) were positive, defined by the presence of at least one punctate signal per cell (Figure 2B). Based on these criteria, *KDM5D* RNAscope analysis achieved a sensitivity of 100% (9/9) and a specificity of 76.2% (64/84) for the identification of LOY in meningioma samples.

### Clinicopathological features of LOY meningioma

**Figure 3:**
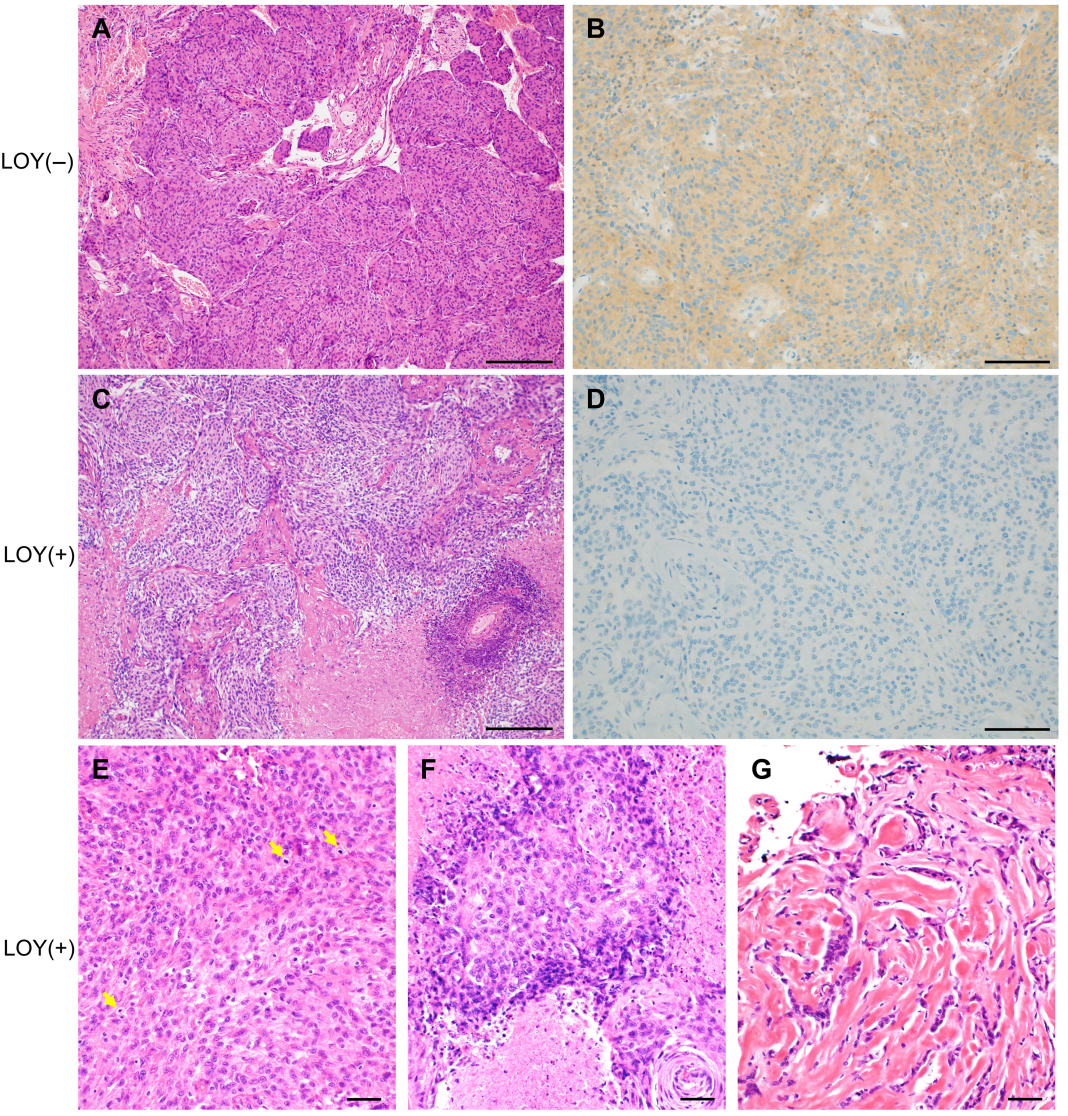
Histopathological features associated with Y chromosome loss in meningiomas. (A) H&E staining of representative LOY‐negative meningioma, classified as CNS WHO grade 1. Scale bar, 100 μm. (B) Merlin immunohistochemistry showing diffuse cytoplasmic expression in a representative LOY‐negative meningioma, classified as CNS WHO grade 1. Scale bar, 200 μm. (C) H&E staining of a representative LOY meningioma, classified as CNS WHO grade 2. Scale bar, 100 μm. (D) Merlin immunohistochemistry of the LOY meningioma, classified as CNS WHO grade 2, showing loss of merlin expression. Scale bar, 200 μm. (E) LOY meningiomas, classified as CNS WHO grade 2, displaying a sheet‐like architecture with frequent mitotic figures (arrows). Scale bar, 50 μm. (F) LOY meningiomas, classified as CNS WHO grade 2, with spontaneous necrosis. Scale bar, 50 μm. (G) LOY meningiomas, classified as CNS WHO grade 2, exhibiting prominent dense collagen fiber bundles. Scale bar, 50 μm.

No significant differences in age between patients with LOY and non‐LOY meningiomas were noted (mean age: 67 versus 67 years; *p* = 0.373). To characterize the molecular features of LOY meningiomas, immunohistochemistry and Sanger sequencing were performed. Loss of merlin expression was observed in 88.9% (8/9) of LOY meningiomas, compared to 50.0% (42/84) of non‐LOY meningiomas (*p* = 0.035) (Table 1, Figure 3A–D). Cytoplasmic MTAP staining, used as a surrogate marker for *CDKN2A/B* status, was retained in all samples. No *TERT* promoter mutations were detected in any case. Among LOY meningiomas, 44.4% (4/9) were classified as WHO grade 1% and 55.6% (5/9) as WHO grade 2. In contrast, among non‐LOY meningiomas, 85.7% (72/84) were grade 1% and 14.3% (12/84) were grade 2 (Table 1). LOY status was significantly associated with a higher WHO grade (*p* = 0.009). There was no significant difference in the Ki‐67 labeling index between the two groups. Among WHO grade 2 meningiomas, significant differences were observed in histopathological features, with LOY meningiomas more frequently exhibiting spontaneous necrosis and sheeting compared to non‐LOY counterparts (Table 2, Figure 3E,F, *p* = 0.003, *p* = 0.015, respectively). In addition, dense collagen fiber bundles were more commonly observed in LOY meningiomas (Table 2, Figure 3E,G, *p* < 0.001).

**Table 2:** Histopathological features of LOY meningiomas.

	LOY(+)	LOY(−)	*p* value
Increased cellularity	7/9 (77.8)	37/84 (44.0)	0.079
Small cells with N/C ratio	4/9 (44.4)	23/84 (27.4)	0.439
Prominent nucleoli	2/9 (22.2)	26/84 (31.0)	0.719
Sheeting	4/9 (44.4)	8/84 (9.5)	0.015*
Foci of spontaneous necrosis	4/9 (44.4)	4/84 (4.8)	0.003*
Brain invasion	1/9 (11.1)	3/84 (3.6)	0.339
Dense collagen fiber bundles	7/9 (77.8)	15/84 (17.9)	< 0.001*
Mitosis (/10 HPF of each 0.24 mm^2^)	0 [0, 2.5]	0 [0, 7.5]	0.104

Values are shown as *n* (%) or median [interquartile range (IQR): 25th percentile, 75th percentile].

LOY, loss of Y chromosome; N/C, nuclear‐to‐cytoplasmic; HPF, high‐power field.

* *p* < 0.05.

**Figure 4:**
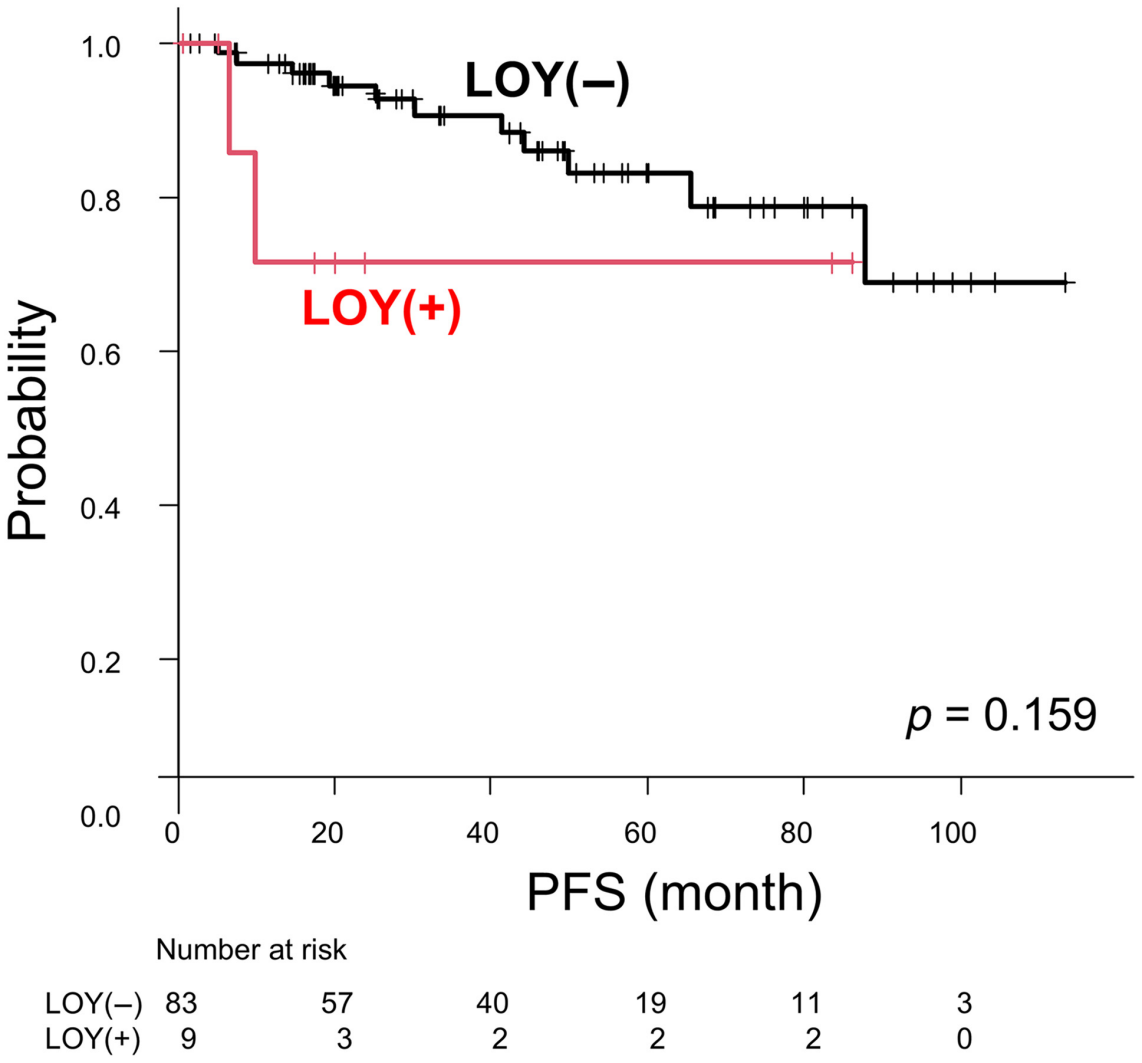
Kaplan–Meier survival curves for PFS in patients with LOY and non‐LOY meningiomas.

The 5‐year PFS rate for the entire cohort was 81.9% [95% confidence interval (CI): 68.8–89.9%]. When stratified by LOY status, the 5‐year PFS rates were 83.0% (95% CI: 69.1–91.1%) for non‐LOY meningiomas and 71.4% (95% CI: 25.8–92.0%) for LOY meningiomas (Figure 4, *p* = 0.159). Stratification by WHO grade revealed a 5‐year PFS rate of 57.4% (95% CI: 19.3–82.9%) for grade 2 meningiomas and 85.9% (95% CI: 71.7–93.3%) for grade 1 meningiomas, with grade 2 tumors exhibiting significantly shorter PFS (*p* = 0.00462; supplementary material, Figure S2A). Merlin expression status was not significantly associated with clinical outcomes (*p* = 0.547 for PFS; supplementary material, Figure S2B).

### Characterization of LOY meningioma by spatial transcriptome analysis

Three LOY and six non‐LOY meningioma cases were subjected to the 10x Genomics Xenium platform. A total of 337,566 cells were obtained from eight cases, with one LOY case excluded during data filtering and quality control. The omitted LOY case (No. 2) was characterized by a median gene count of 123 and a median transcript count of 89.

**Figure 5:**
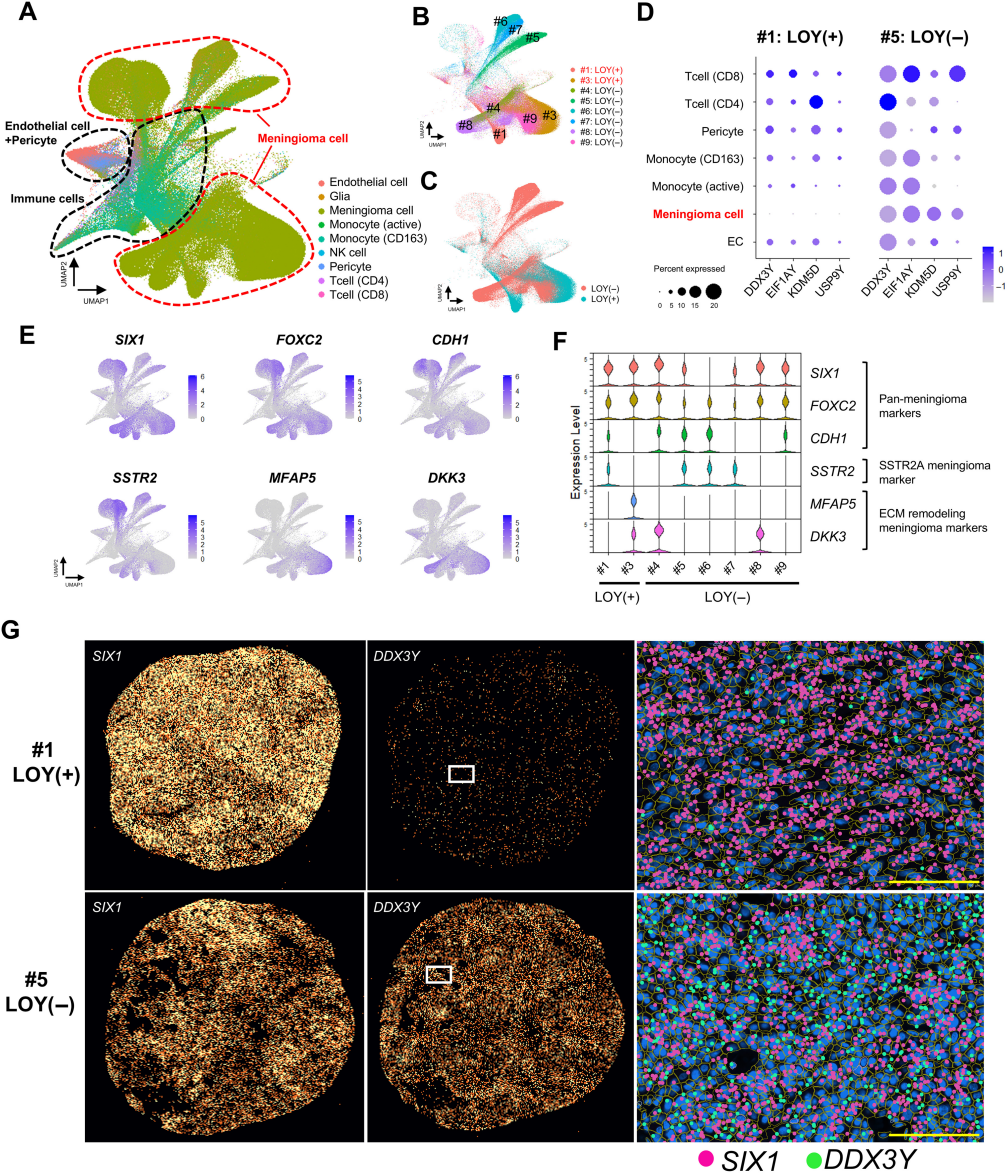
Spatial transcriptomic profiling of LOY and non‐LOY meningiomas. (A) UMAP plot displaying nine distinct clusters in male meningiomas. (B) UMAP plot illustrating case‐wise clustering patterns across individual meningioma samples. (C) UMAP visualization indicating distribution of LOY‐positive (LOY+) and LOY‐negative (LOY−) clusters. (D) Dot plot of Y chromosome gene expression in LOY+ and LOY− meningiomas. Dot size denotes percentage of cells expressing the gene; color intensity reflects the average expression level. (E) Feature plots exhibiting scaled RNA expression of selected genes across annotated cell types in UMAP space. *SIX1*, *FOXC2*, and *CDH1* are broadly expressed in meningioma cells, whereas *SSTR2*, *MFAP5*, and *DKK3* display restricted expression in specific meningioma subpopulations. (F) Violin plots depicting the expression distributions of *SIX1*, *FOXC2*, *CDH1*, *SSTR2*, *MFAP5*, and *DKK3* across individual meningioma cases. (G) Xenium spatial transcriptomics analysis of two representative cases – LOY meningioma (No. 1) and non‐LOY meningioma (No. 5). Left: spatial distribution of *SIX1*, a pan‐meningioma marker. Middle: expression of *DDX3Y*, a Y‐linked gene. Right: dot plot showing cellular‐level expression of *SIX1* and *DDX3Y*. Scale bar, 100 μm.

Cell clustering analysis identified 49 distinct cell clusters (supplementary material, Figure S3A). Deconvolution using reference single‐cell RNA sequencing data from previously published meningioma studies enabled the identification of nine major cell types, including meningioma cells, immune cells, endothelial cells, and pericytes (Figure 5A) [23]. UMAP revealed that cells from each case were largely grouped, and tumor cells from LOY cases showed a tendency to cluster, as distinct from non‐LOY cases (Figure 5B,C).

To evaluate LOY status, four Y chromosome‐encoded genes – *DDX3Y*, *EIF1AY*, *KDM5D*, and *USP9Y* – were selected from the eight Y‐linked genes included in the Xenium 5 K panel, based on their consistent and robust signal intensity. In LOY tumors, expression of all four genes was specifically lost in meningioma cells but retained in adjacent nontumor cells (Figure 5D; supplementary material, Figure S3B). Our dataset further identified *SIX1*, *FOXC2*, and *CDH1* as pan‐meningioma markers, exhibiting consistent expression across tumor cells irrespective of LOY status (Figure 5E; supplementary material, Figure S3C,D) [32]. In contrast, markers such as *SSTR2*, *MFAP5*, and *DKK3*, which define meningioma tumor cell subtypes, exhibited mutually exclusive expression patterns that were independent of LOY status (Figure 5E,F; supplementary material, Figure S4). High‐resolution spatial mapping of the tumor microenvironment further demonstrated that *SIX1* expression remained consistent across samples, while *DDX3Y —* a representative Y chromosome marker — was significantly downregulated in LOY meningioma tumor cells compared to non‐LOY counterparts (Figure 5G).

**Figure 6:**
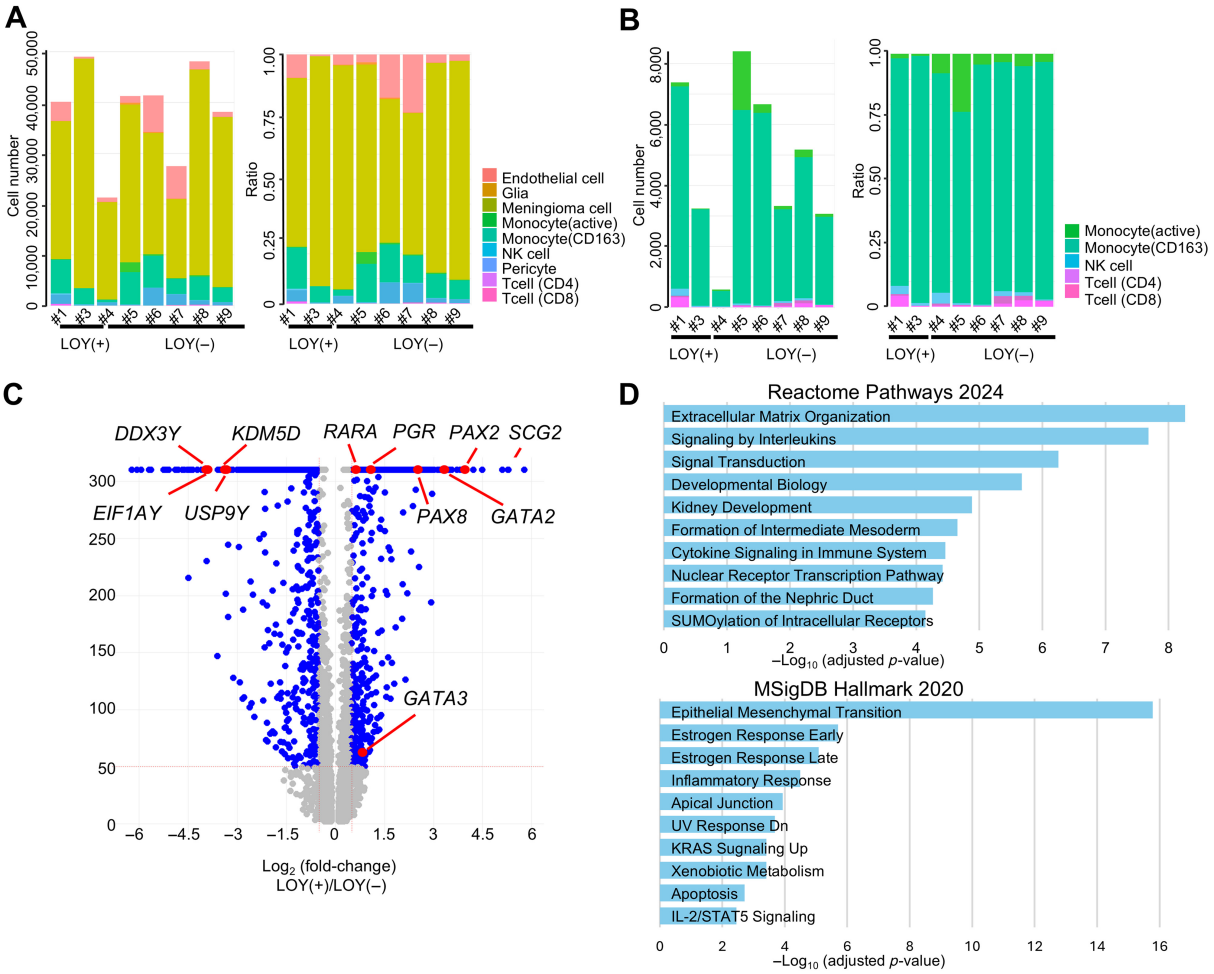
Cellular composition and transcriptional alterations associated with LOY. (A) Total cell counts and relative proportions of meningioma cells across cases. (B) Quantification and proportional distribution of meningioma‐associated immune cells in each case. (C) Volcano plot of differentially expressed genes (DEGs) between LOY and non‐LOY clusters. Notably, Y‐linked genes *DDX3Y*, *EIF1AY*, *KDM5D*, and *USP9Y* were downregulated in LOY meningiomas. (D) Gene set enrichment analysis (GSEA) of DEGs between LOY and non‐LOY clusters. Enrichment was evaluated using Reactome Pathways 2024 and MSigDB Hallmark 2020 datasets, with significance indicated as −log₁₀(*p* value).

The proportion of meningioma tumor cells among all cell types ranged from 57% (No. 7) to 92% (No. 3) (Figure 6A). Macrophages (monocytes) represented the most abundant non–tumor cell population, and the composition and relative abundance of immune cell types were comparable between LOY and non‐LOY meningiomas (Figure 6B). Differential gene expression analysis revealed a distinct transcriptional profile, including upregulation of *RARA*, *PGR*, *BMPR1B*, *PAX8*, *GATA2*, *PAX2*, *SCG2*, and *GATA3* in LOY meningioma (Figure 6C). Gene set enrichment analysis of 485 differentially expressed genes (log₂FC > 0.5; −log₁₀(*p* value) > 50) indicated that LOY meningiomas exhibit transcriptional alterations associated with key tumor‐related biological processes, including ‘epithelial–mesenchymal transition’ and ‘extracellular matrix organization’ (Figure 6D).

## Discussion

We identified LOY in 9.7% (9/93) of tumor tissue DNA samples from 85 male patients with meningioma using ddPCR for screening and MLPA for validation. RNAscope analysis of *KDM5D* expression demonstrated high sensitivity for detecting LOY in FFPE sections. LOY was significantly associated with a higher WHO tumor grade (*p* = 0.009) and increased frequency of merlin loss (*p* = 0.035).

LOY is a well‐established physiological, age‐related event in PBCs, with prevalence increasing with age, affecting approximately 7%, 14%, 18%, and 20% of men aged < 65, 66–75, 76–85, and ≥ 86 years, respectively [5, 33]. Despite the widespread implementation of comprehensive genomic profiling in various malignancies, LOY in tumor cells has received relatively little attention [34]. This underrepresentation stems from several technical challenges, including the small number of protein‐coding genes on the Y chromosome, its haploid nature, extensive homology with regions of the X chromosome, and the presence of repetitive sequences resulting from gene amplification [12]. In addition, the inclusion of Y chromosome gene expression data in unsupervised clustering analyses inherently stratifies samples by sex, which may lead researchers to exclude Y chromosome‐related signals, to avoid sex‐based segregation when characterizing the broader genomic landscape of tumors.

Previous studies by Sayagues *et al* and Domingues *et al* used interphase FISH to investigate numerical chromosomal aberrations in meningiomas, reporting LOY in 32% (32/100) and 26% (24/91) of cases, respectively, with abnormalities in chromosome 22 being the most frequently observed alterations [35, 36]. However, FISH is limited in sensitivity and specificity for detecting gene deletions, primarily due to its dependence on interpreting three‐dimensional nuclear structures in a two‐dimensional imaging plane and the inherent constraints of dark‐field microscopy. Although next‐generation sequencing (NGS) methodologies frequently omit sex chromosomes from analysis, our previous whole‐exome sequencing of a sporadic case of multiple male meningiomas revealed LOY co‐occurring with chromosome 22 loss as recurrent genomic events [13]. In a separate study using whole‐exome sequencing of 169 meningioma samples from 53 patients with progressive or high‐grade tumors, somatic LOY was identified in 64% (9/14) of male patients, emphasizing its potential relevance in the pathogenesis of aggressive meningioma [37].

Our study represents the first application of ddPCR combined with MLPA to evaluate LOY status in tumor tissue DNA from meningioma patients. ddPCR is a simple, highly sensitive, and cost‐effective technique for detecting copy‐number aberration in FFPE‐derived DNA and has been previously used to detect LOY in peripheral blood and kidney tissues [38, 39]. MLPA provides complementary advantages, enabling the detection of DNA copy‐number alterations across approximately 50 loci within a single semiquantitative assay, thereby overcoming the locus‐specific limitations of ddPCR, which can evaluate only a limited subset of Y‐linked and reference genes. In this study, we employed ddPCR for coarse screening and subsequently extracted complete LOY using MLPA. ddPCR‐based screening targeting *SRY* proved effective, as deletion involving *SRY* generally corresponded to complete LOY, with only a few exceptions. In addition, we demonstrated that RNAscope analysis provides high sensitivity for LOY detection and represents a valuable alternative in cases with low tumor content or where DNA extraction is not feasible. Importantly, RNAscope enables direct visualization of Y‐linked gene expression at RNA level within tumor cells on tissue section and facilitates integration with spatial information, offering enhanced utility for spatially resolved transcriptomic analysis.

Spatial transcriptome analysis revealed LOY at the single‐cell level and further delineated the transcriptional landscape of LOY meningiomas. LOY‐linked gene expression was confined to tumor cells, whereas expression was preserved in surrounding nontumor cells. Pathway enrichment analysis indicated that LOY may contribute to tumor aggressiveness through activation of the epithelial–mesenchymal transition pathway. Furthermore, extracellular matrix organization emerged as a hallmark biological feature of LOY meningiomas, supported by histological evidence of dense collagen fiber bundles. A previous study using photon fluorescence endomicroscopy, which capitalizes on the endogenous fluorescence of tissue components, demonstrated that WHO grade 2 meningiomas are typified by prominent, block‐like perivascular and interstitial collagen deposits, which may coalesce into extensive acellular zones, features less commonly observed in grade 1 tumors [40].

We identified a significant association between LOY in meningiomas, a higher WHO tumor grade, and loss of merlin protein expression. Histopathological analysis revealed that LOY was significantly associated with key morphological features of malignancy, including sheeting architecture and spontaneous necrosis [2]. Notably, LOY was frequently observed in primary atypical (WHO grade 2) meningiomas with loss of merlin, indicating that it may represent a trunk genetic event occurring in parallel with *NF2* alterations during early tumorigenesis. This finding is consistent with our previous study, in which concurrent copy‐number alterations on chromosomes 22q and Y were recurrent early events in tumor development [13].

Our study has several limitations. First, the relatively small cohort, analyzed using the Xenium platform, and the absence of a comprehensive methodological framework to assess the functional status of Y‐chromosome genes limit the interpretation of their biological roles in LOY meningiomas. Future experimental strategies, such as LOY modeling, are needed to clarify the functional role of LOY in meningioma biology. Second, the relatively short follow‐up period and limited sample size may have underestimated the potential association between LOY and adverse prognostic outcomes in male patients with meningiomas. Long‐term, multicenter studies are essential to better characterize the clinical behavior of LOY meningiomas.

In conclusion, we used ddPCR in combination with MLPA for the first time to evaluate LOY status in male meningiomas, revealing a significant prevalence of LOY and implicating its potential contribution to a more aggressive histopathological phenotype compared to non‐LOY meningiomas.

## Author contributions statement

MS, MH and DM conceived and designed the study. MS and MH conducted the experiments and contributed to the analysis of sequencing and imaging data. ST and MN coordinated sample acquisition. MS and HI performed tissue preparation and histopathological evaluation. MS, MH, AY and DM interpreted the findings and supervised the research. MS, MH and DM drafted the manuscript. All authors reviewed and approved the final version of the manuscript.

## Supporting information


**Figure S1.** MLPA results in cases with and without complete loss of chromosome Y
**Figure S2.** Prognostic impact of WHO grade and merlin expression
**Figure S3**. Extended clustering and gene expression patterns in spatial transcriptomics
**Figure S4**. Spatial transcriptomic maps showing expression patterns of *SIX1*, *SSTR2*, *MFAP5*, and *DKK3*

**Table S1.** P360 Y‐chromosome probemix B2

## Data Availability

The data that support the findings of this study are available from the corresponding author upon reasonable request.
