# Supplementary material for: Clinicopathological significance of loss of Y chromosome in male meningiomas
Source: J Pathol. 2026 Feb 19;269(1):125–37. doi: 10.1002/path.70040 (PMC13050807; doi:10.1002/path.70040)
Supplement: Supplementary file 1 — Figure S1. MLPA results in cases with and without complete loss of chromosome Y Figure S2. Prognostic impact of WHO grade and merlin expression Figure S3. Extended clustering and gene expression patterns in spatial transcriptomics Figure S4. Spatial transcriptomic maps showing expression patterns of SIX1, SSTR2, MFAP5, and DKK3 [file PATH-269-125-s002.docx]

**Clinicopathological significance of loss of Y chromosome in male meningiomas**

M Sakaguchi *et al. J Pathol* <https://doi.org/10.1002/path.70040>

**Supplementary Figures S1–S4**

**Supplementary Table S1 (provided as a separate Excel file)**


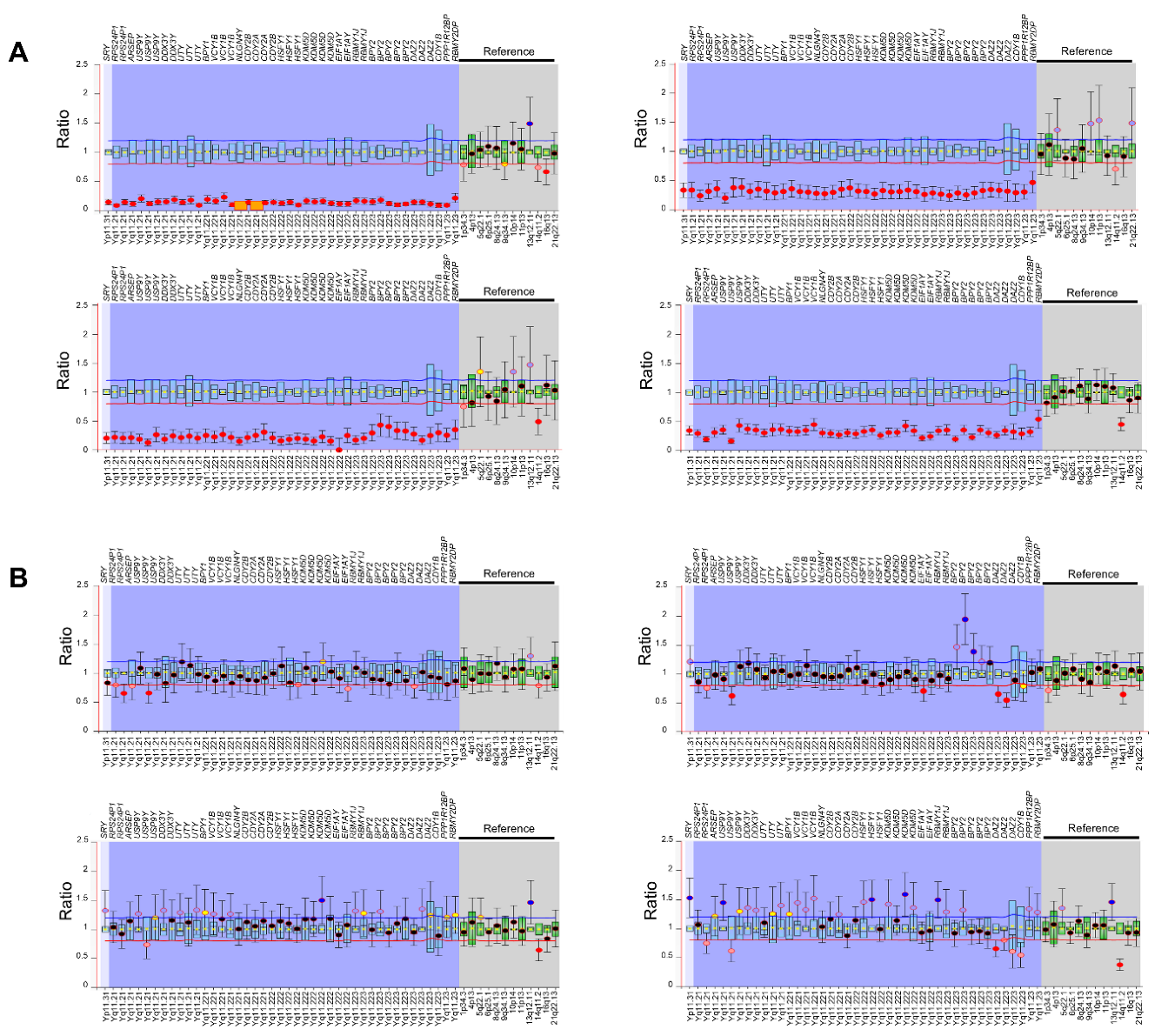


**Figure S1. MLPA results in cases with and without complete LOY.** (A) Representative MLPA results for four additional cases exhibiting complete LOY. (B) Representative MLPA results for four additional cases without complete LOY.


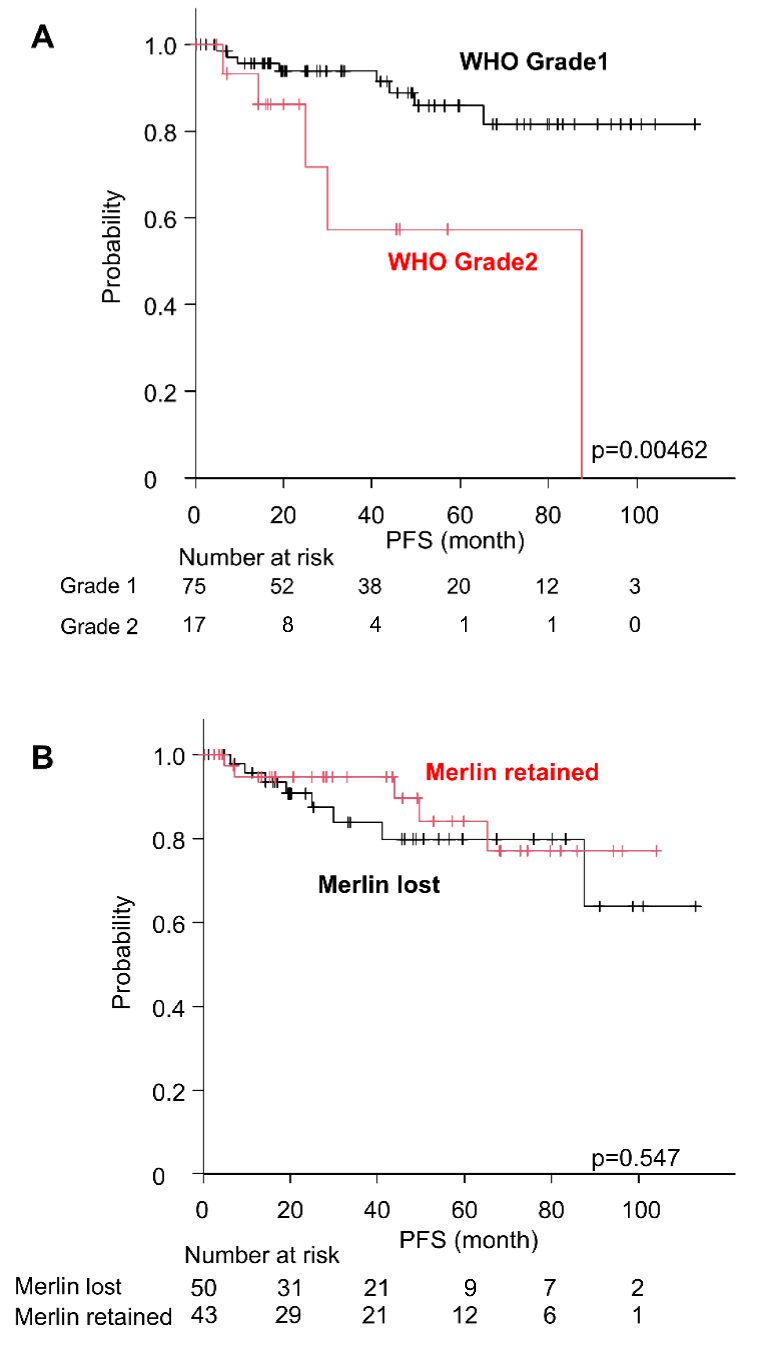


**Figure S2. Prognostic impact of WHO grade and merlin expression.** (A) Kaplan–Meier survival curves for PFS stratified by WHO grade 1 and grade 2 meningiomas. (B) Kaplan–Meier survival curves comparing PFS in meningiomas with merlin loss versus merlin retention.


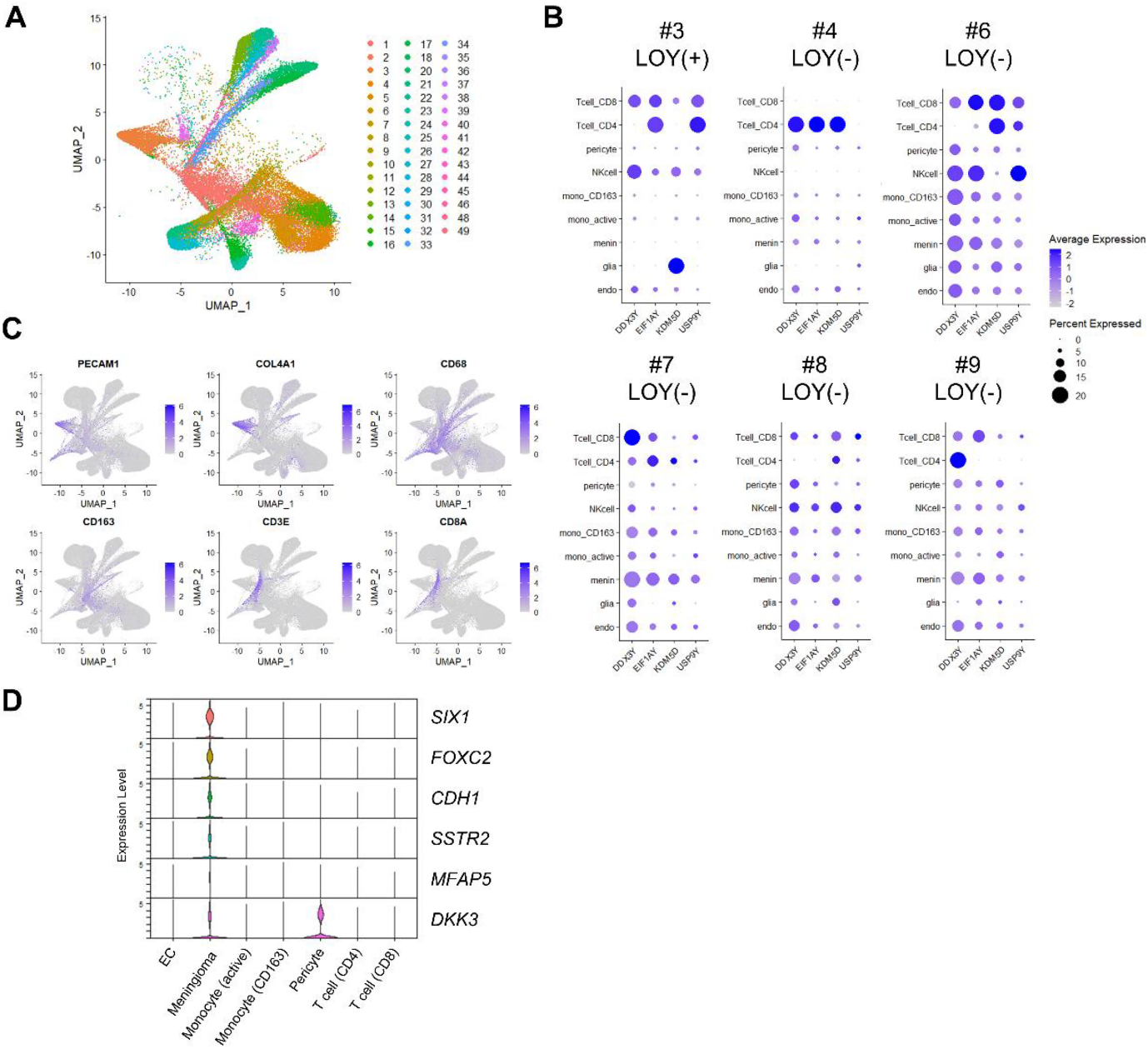


**Figure S3. Extended clustering and gene expression patterns in spatial transcriptomics.** (A) UMAP visualization displaying 49 clusters identified in male meningiomas. (B) Dot plot of Y chromosome gene expression across meningioma cases. Dot size indicates the proportion of cells with detectable transcript expression; color intensity reflects average gene expression levels. (C) Feature plots displaying scaled RNA expression across annotated nontumor cell types in UMAP space. (D) Violin plots showing the expression levels of *SIX1*, *FOXC2*, *CDH1*, *SSTR2*, *MFAP5*, and *DKK3* at the single-cell level.


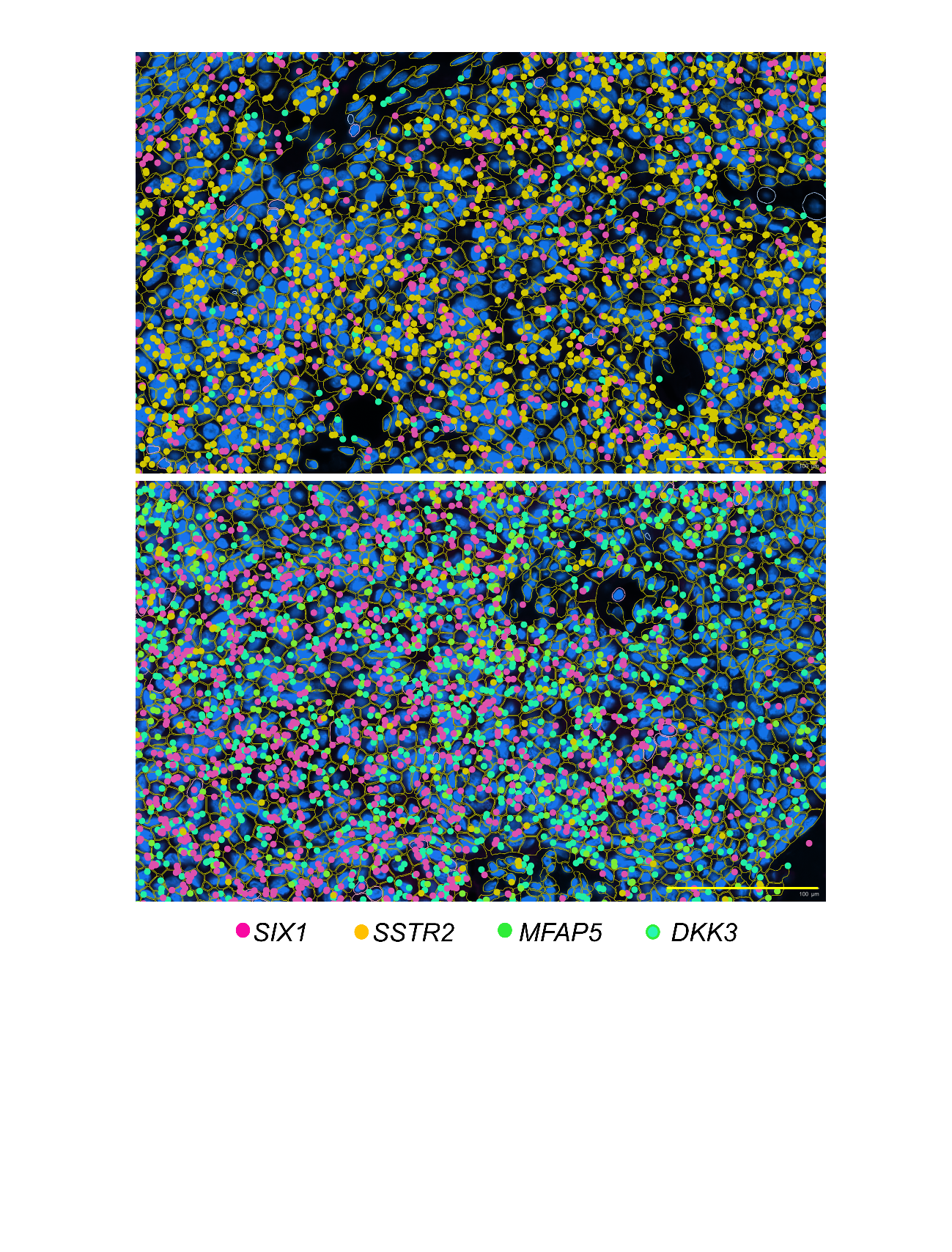
**Figure S4. Spatial transcriptomic maps showing expression patterns of *SIX1*, *SSTR2*, *MFAP5*, and *DKK3*.** The upper panel emphasizes predominant *SSTR2* expression, whereas the lower panel illustrates *MFAP5* and *DKK3* enrichment in spatially distinct regions in non-LOY case (No. 5). Scale bar, 100 µm.
